# Low expression of PTK6/Brk predicts poor prognosis in patients with laryngeal squamous cell carcinoma

**DOI:** 10.1186/1479-5876-11-59

**Published:** 2013-03-07

**Authors:** Xue-Kui Liu, Xin-Rui Zhang, Qian Zhong, Man-Zhi Li, Zhi-Min Liu, Zhi-Rui Lin, Di Wu, Mu-Sheng Zeng

**Affiliations:** 1 State Key Laboratory of Oncology in South China, Sun Yat-Sen University Cancer Centre, Guangzhou, Guangdong, China; 2 Department of Head and Neck Surgery, Sun Yat-Sen University Cancer Centre, Guangzhou, Guangdong, China; 3 Department of Experimental Research, Sun Yat-Sen University Cancer Centre, Guangzhou, Guangdong, China; 4 Sun Yat-sen University Cancer Center, 651 Dongfeng Road East, Guangzhou, 510060, China

**Keywords:** PTK6/Brk, Laryngeal squamous cell carcinoma, Prognosis

## Abstract

**Background:**

Protein tyrosine kinase 6 (PTK6), also known as breast tumor kinase (Brk), was a nonreceptor tyrosine kinase containing SH3, SH2, and tyrosine kinase catalytic domains. The deregulated expression of PTK6 was observed in various human cancers. However, little was known about PTK6 expression and its clinicopathological significance in human laryngeal squamous cell carcinoma (LSCC).

**Materials:**

PTK6 expression was evaluated in 7 pairs of surgically resectable laryngeal tissues by Western blotting and in 13 pairs of surgically resectable laryngeal tissues by reverse transcription-PCR (RT-PCR). Using immunohistochemistry, we performed a retrospective study of the PTK6 expression levels on 134 archival LSCC paraffin-embedded samples. Prognostic outcomes correlated with PTK6 were examined using Kaplan–Meier analysis and Cox proportional hazards model.

**Results:**

The PTK6 expression level was lower in LSCC tissues than in the adjacent noncancerous epithelial laryngeal tissues by Western blots and RT-PCR. By immunohistochemical analysis, we observed high expression of PTK6 in 25 of 76 (32.9%) adjacent noncancerous epithelial laryngeal tissues and in 39 of 134 (29.1%) of LSCC, respectively. Multivariate analysis demonstrated that pN status and the expression level of PTK6 (*P* < 0.05) were independent and significant prognostic factors. In the primary LSCC category, median DFS (disease free survival) of high, medium and low PTK6 expression patients were 88.5 months ,74.5 months and 49.0 months (log-rank test, *P* = 0.002); median OS (overall survival) of high, medium and low PTK6 expression patients were 88.5 months ,76.3 months and 65.7 months (log-rank test, *P* = 0.002). Reduced cytoplasmic PTK6 expression in LSCC was significantly associated with late pN status (*P* =0.005, r = 0.27), advanced pTNM stages (III and IV) (*P* =0.027, r = 0.147), and poor differentiated LSCC (*P* <0.0001, r = 0.486). In adjacent paracancerous laryngeal epithelial samples, median DFS of high, medium and low PTK6 expression patients were 92.6 months ,75.6 months and 48.5 months (log-rank test, *P* = 0.020); median OS of high, medium and low PTK6 expression patients were 92.9 months ,78.9 months and 74.6 months (log-rank test, *P* = 0.042).

**Conclusion:**

The present findings indicated that cytoplasmic PTK6 expression is a potential prognostic factor for survival in LSCC patients. High expression of PTK6 was associated with favorable OS and DFS in LSCC patients.

## Background

The cytoplasmic non-receptor tyrosine kinase PTK6 (BRK, breast tumour kinase) was originally cloned from a human metastatic breast tumour [1]. The 53 kDa PTK6 protein was a cytoplasmic tyrosine kinase, and structurally resembled tyrosine kinases of the Src family [2]. Src family tyrosine kinases play important roles in epithelial tumor development. Therefore, it was proposed that PTK6 may play a role in epithelial tumorigenesis [3,4].

The overexpression of tyrosine kinases (including EphA1, PTK6/BRK, and Ron) were reported in head and neck cancers which included pharyngeal, hypopharyngeal, tonsilar, supraglottic and some oral cancers [5]. However, PTK6 expression in tumor tissues was complex. For example, PTK6 was localized in the nucleus and cytoplasm of normal oral epithelium, and in perinuclear regions of poorly differentiated oral squamous carcinomas [3]. PTK6 expression was low or undetectable in normal ovary [2] or normal breast epithelium [3,4,6], but it was found in human ovarian tumor cells [2] and in greater than 60% of breast tumors and breast cancer derived cell lines [7], indicating that overexpression of PTK6 may be related to carcinogenesis. Aubele et al. [6] reported that PTK6 protein expression had prognostic value in a small set of 105 breast carcinomas. However, the expression of PTK6 and its clinical significance were not clearly documented in LSCC. In the current study, we aimed to investigate PTK6 expression and analyze its association with clinicopathological factors to understand its potential role in LSCC.

## Material and method

### Tissue samples and patients

Fresh tumor tissue samples with paired non-cancerous normal mucosa (with more than a 5-mm distance from the primary tumor’s edge) of 13 LSCC patients were obtained at the time of operation from the Sun Yat-sen University Cancer Center (SYSUCC). All the 13 patients were histologically confirmed as LSCC by biopsy pre-operation and all these patients underwent total laryngectomy in the primary site. Before making extraction, we did frozen section and made sure that all the specimens were done on at least 70%-80% tumor cells under the microscope. A total of 134 patients who were previously untreated and histologically confirmed LSCC, in resectable stages (T1~4aN0-3 M0 ) (Union for International Cancer Control ,UICC 2002) were eligible between January 2003 and December 2005 at SYSUCC in our study. Patients who had previous malignant disease, a second primary tumor, positive margin, or died of postoperative complications were excluded. Pretreatment evaluations included a complete history, physical examination, performance status, serum chemistry profile, complete blood cell count, chest radiography, computed tomography (CT) or magnetic resonance imaging (MRI), bilateral cervical and supraclavicular ultrasonography, and abdominal ultrasonography. The medical records of these patients were reviewed to assess the patients’ characteristics, including age, sex, primary site, clinical stage, date of disease progression, and final status on the last follow-up examination. The informed consent was obtained from each patient prior to surgery and the study was approved from the Institute Research Ethics Committee.

### Western blot

Western blotting analysis was carried out with the proteins collected from the adjacent normal epithelium tissues and the cancer tissues, and total proteins were extracted with 1 X sodium dodecyl sulfate (SDS) sample buffer (2% SDS, 62.5 mmol/L Tris–HCl (pH 6.8), 5% 2-mercaptoethanol, and 10% glycerol) . The concentration of the protein was measured by the BCA protein assay kit (PIERCE, Rockford, IL, USA). A total of 20 μg protein was electrophoretically separated in 12% SDS polyacrylamide gels and transferred onto polyvinylidene difluoride membranes (Amersham Pharmacia Biotech, Piscataway, NJ). Then incubate the primary polyclonal antibody against PTK6 (dilution, 1:1000; Abgent INC, USA),which was mixed with PTK6 Antibody (N-term) Blocking Peptide (0.25 ug/ml ), in 5% of the skimmed milk solution overnight at 4°C, and anti-rabbit (1:3000; Santa Cruz Biotechnology, Santa Cruz, CA) secondary antibody was were used to detect PTK6 protein. Anti-glyceraldehyde 3-phosphate dehydrogenase (GAPDH) (1:4000, Santa Cruz, CA, USA) antibody and anti-mouse (1:4000; Santa Cruz Biotechnology, Santa Cruz, CA) secondary antibody were used to confirm equal loading. The protein signals were detected by the enhanced chemiluminescence (ECL) detection system (Amersham Biosciences Europe, Freiburg, Germany) according to the manufacturer's protocols.

### Real time reverse transcription-polymerase chain reaction (RT-PCR) analysis

Total RNAs from fresh tissues were purified from tissues using TRIzol Reagent (Invitrogen, Carlsbad, CA, USA) according to the manufacturer’s instructions, and 2 μg RNA of each sample was reverse transcribed using SuperScript RT kit (Invitrogen Life Technologies, Carlsbad, CA, USA). Full-length open reading frame of PTK6 was amplified by PCR from cDNA samples of normal laryngeal epithelium tissues and laryngeal carcinoma tissues. Real-time PCR was carried out using a CFX96 Real-Time System (BIO-RAD) (PREMIER Biosoft International, Palo Alto CA, USA). Sequences of the primers were as follows: PTK6, 5’-TACTTTGGGGAGGT CTTCGAG-3’(sense), 5’-TGCCGCAGCTTCTTCATG-3’(antisense);GAPDH, 5'-GACTCATGACCACAGTCCATGC-3' (sense), 5'-AGAGGCAGGGATGATGTTCTG-3' (antisense). We used the SYBR Green kit (Invitrogen Life Technologies, Carlsbad, CA, USA) to execute the amplification of the cDNA. The RT-PCR cycling parameters were performed as follows: denaturation at 95°C for 15 seconds, annealing at 55°C for 30 seconds, and extension at 72°C for 30 seconds. The expression data were normalized to the geometric mean of housekeeping gene GAPDH to control the difference in expression levels and analyzed using the 2-Delta Delta C (T) method described by the previous report [8].

### Immunohistochemistry

Immunohistochemistry was performed to examine the PTK6 expression in 134 LSCC tissue specimens. PTK6 was detected using a mouse monoclonal antibody against PTK6 (Abgent INC, USA). Briefly, paraffin-embedded specimens were cut into 4 μm sections and baked at 65°C for 30 minutes, and a paraffin section of the LSCC tissue from the patients was deparaffinized with xylene and rehydrated through graded alcohol. Antigenic retrieval was processed by submerging the sample in citrate buffer (pH 6) and microwaving for 4 minutes, following by submergence with 3% hydrogen peroxide to quench the activity endogenous peroxidase for 15 minutes. The tissue slides were incubated with anti-PTK6 antibody at a dilution of 1:150 for 12 hours at 4°C in a moist chamber, and the anti-PTK6 antibody was mixed with PTK6 Antibody (N-term) Blocking Peptide ( 2.5 ug/ml ). Subsequently, biotinylated anti-rabbit secondary antibody was applied for 35 minutes at 37°C. Then, the sections were incubated with streptavidin–horseradish peroxidase complex and developed with 3-diaminobenzidine tetrahydrochloride (DAB) for 2 minutes. Mayer’s hematoxylin was applied as a counterstain using Olympus CX31 microscope (Olympus, Center Valley, PA). RT-PCR and immunohistochemistry were performed to examine the PTK6 expression in 10 invasive ductal breast cancer tissue specimens, which revealed high or strong expression of PTK6 at both mRNA and protein levels in 7 cases, and thus they were used as positive controls. Normal rabbit serum was served as a negative control.

Cytoplasmic PTK6 was evaluated according to the percentage of stained cells (median, 60%; range, 0 to 100%) and staining intensity: negative staining; low staining, light yellow; intermediate staining, yellow brown; and high staining, brown. The proportion of immunopositive cells(categorized as follows: 0, <10%; 1, ≥10% to <25%; 2, ≥25% to <50%; 3, ≥50% to <75%; and 4, ≥75%)and the intensity of immunostaining (0,absent; 1,weak; 2, moderate; 3,strong), as described [9]. The final scores were the sum of the two sides ranged from 0 to 7. According to biological behavior of squamous cell carcinoma, all LSCC patients were divided into three groups (1–2, low expression; 3–5, medium expression and 6–7, high PTK6 expressing). Each section was evaluated by two independent pathologists without knowledge of the clinical features of the cases.

### Statistical analysis

The paired *T*-test was used to analyze the significance of PTK6 protein levels and mRNA levels in the paired samples. The chi-square test was employed to evaluate differences of PTK6 expression between normal laryngeal epithelium tissues and LSCC tissues. The chi-square test or Fisher’s exact test was used to analyze the relationship between the PTK6 expression and clinicopathological factors. Kaplan–Meier and log-rank tests were used for survival analysis. Multivariate Cox regression analysis was performed for all variables that were found to be significant by univariate analysis. *P* values less than 0.05 were considered statistically significant. The SPSS statistical software package version 16.0 (SPSS Inc., Chicago, IL, USA) was used for all analyses. The survival time was defined as from the date of surgery to the date of death or final clinical follow-up (July, 2012), respectively.

## Result

### Expression of PTK6 in LSCC tissues by western blotting

**Figure 1:**
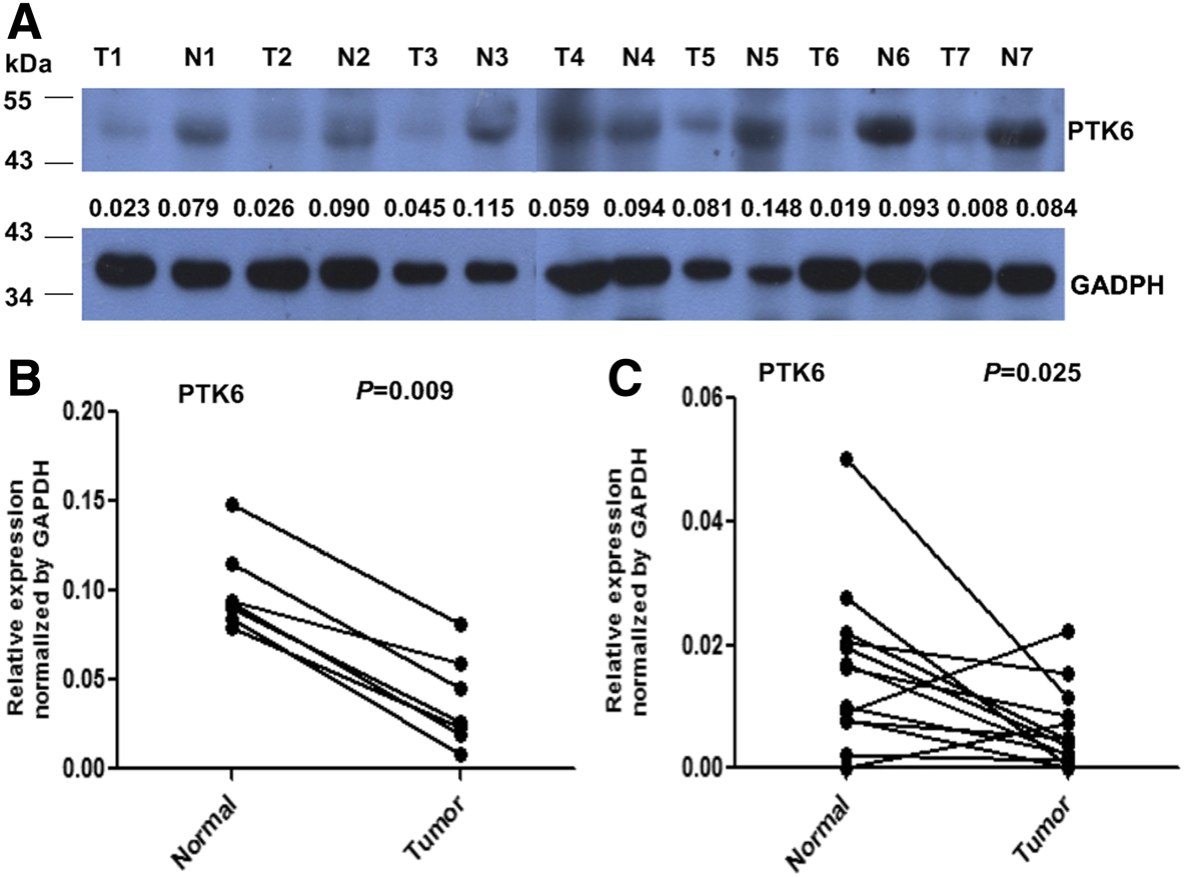
**Expression levels of PTK6 in laryngeal squamous cell carcinoma tissues. A**. Expression levels and quantitative analysis of PTK6 protein in 7 paired laryngeal squamous cell carcinoma tissues by Western blotting. N: paracarcinoma (normal) laryngeal epithelial tissues. T: laryngeal squamous cell carcinoma tissues. The numbers under each PTK6 lane indicated relative expression of each sample by normalizing to GADPH which serves as an internal control. **B**: The relative quantity of PTK6 protein was determined by densitometric analysis of the Western blots. **C**: mRNA level of PTK6 in 13 paired laryngeal squamous cell carcinoma tissues by real-time PCR. N: paracarcinoma (normal) laryngeal epithelial tissues. T: laryngeal squamous cell carcinoma tissues.

We examined PTK6 protein expression in 7 pairs of LSCC tissues and the paracancerous epithelium tissues by Western blotting. As shown in Figure 1A, the expression level of PTK6 was lower in LSCC tissues than that in the paired noncancerous tissues, though the expression level of PTK6 was variable among different pairs of laryngeal tissues. The relative quantity levels by densitometric analysis showed that PTK6 expression was significantly decreased in LSCC in comparison to the paired noncancerous tissues (Figure 1B).

### Expression of PTK6 in LSCC tissues by RT-PCR

To investigate whether the expression of PTK6 was also reduced in LSCC at mRNA level, we obtained 13 paired LSCC samples to detect PTK6 expression by RT-PCR analysis. As shown in Figure 1C, the reduced PTK6 mRNA expression in LSCC was observed in 11 of the 13 cases, suggesting that the mRNA level of PTK6 was significantly lower in tumor tissues than in paracancerous epithelium tissues.

### Expression of PTK6 in LSCC tissues by Immunohistochemistry

**Figure 2:**
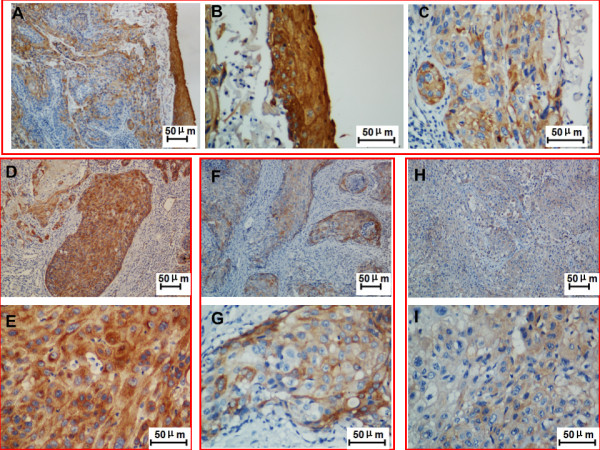
**PTK6 expression in the LSCC tissues by immunohistochemistry analysis. A**. PTK6 protein expression was higher in the normal laryngeal epithelial tissues than in LSCC tissues (original magnification: **A**, x 100); **B **and **C **were higher magnification of part of **A**, **B**. PTK6 immunohistochemical staining in paracarcinoma laryngeal epithelial tissues (original magnification: x 400) **C**. PTK6 immunohistochemical staining in the LSCC epithelial tissues (original magnification:, x 400) ; **D **and **E**. PTK6 immunohistochemical staining in the well differentiated LSCC epithelial tissues (original magnification: **D**, x 100; **E**, x 400); **F **and **G**. PTK6 immunohistochemical staining in the mediate differentiated LSCC epithelial tissues (original magnification: **F**, x100; **G**, x 400); **H **and **I**. PTK6 immunohistochemical staining in the poor differentiated LSCC epithelial tissues (original magnification: **H**, x 100; **I**, x 400).

We further examined the expression of PTK6 protein in 134 paraffin-embedded LSCC samples and 76 matched paracancerous laryngeal epithelial samples by immunohistochemical analysis (Figure 2). The localization of PTK6 in normal laryngeal epithelial and tumor tissue cells displayed a primarily cytoplasmic pattern, while a minority of PTK6 was localized in nucleus. With the same critia for paracancerous laryngeal epithelium and cancer, immunohistochemical analysis showed that high, medium and low expression levels of PTK6 were 25 of 76 (32.9%), 31 of 76 (40.8%) and 20 of 76 (26.3%), respectively, in the whole paracancerous laryngeal epithelial sample, while high, medium and low expression levels of PTK6 were 39 of 134 (29.1%), 59 of 134 (44.0%) and 36 of 134 (26.9%), respectively, in the whole LSCC cohort.

### Correlation of PTK6 expression with clinicopathological characteristics

The median age of total 134 eligible patients was 58 years (range, 25 to 87 years), and 132 cases (98.5%) were men. All 134 patients were surgically treated, 106 patients received no postoperative treatment, 28 patients received adjuvant radiotherapy and 5 patients received adjuvant chemotherapy. Doses of radiotherapy depend on positive surgical margins, number of positive neck nodes, or extracapsular spread. Among 134 LSCC patients, 82(61.2%) received subtotal laryngectomy and 52 (38.8%) underwent total laryngectomy. According to preoperative evaluation, neck dissection and combined with reconstructive surgery was performed if needed.

The relationship between PTK6 expression and different clinicopathological factors was shown in Table 1. Reduced cytoplasmic PTK6 expression in LSCC was found to be associated with late lymph nodal staging (*P* =0.005, r = 0.27), advanced pTNM stages (III and IV) (*P* =0.027, r = 0.147), and poor differentiated LSCC (*P* <0.0001, r = 0.486). However, no correlation was observed between PTK6 expression and other clinicopathologic variables, such as age, sex, pT status, treatment, and tumor location (Pearson’s test, *P* >0.05).

**Table 1:** Expression of PTK6 and Clinicopathologic Characteristics of the Patients With laryngeal Squamous cell carcinoma

**Characteristics**	**Number**	**PTK6 Expression(No)**			**P value**	**r**^**a**^
		**High**	**Medium**	**Low**		
**Age(years)**					0.076	0.193
≤58^b^	69	24	32	13		
>58	65	15	27	23		
**Sex**					0.310	−0.137
male	132	39	57	36		
female	2	0	2	0		
**pT stage**					0.415	0.114
pT1/T2	81	26	32	23		
pT3/T4	53	13	27	13		
**pN stage**					0.005	0.27
pN0	112	36	52	24		
pN+	22	3	7	12		
**pTNM stage**					0.027	0.147
pI/II	72	25	31	16		
pIII/IV	62	14	28	20		
**Treatment**					0.858	−0.099
surgery	101	28	45	28		
surgery+radiotherapy	28	10	12	6		
surgery+chemotherapy	5	1	2	2		
**Pathology grade**					<0.0001	0.486
well	61	29	29	3		
moderately	38	10	15	13		
poorly	35	0	15	20		
**Tumor location**					0.054	0.294
glottic	105	37	46	22		
supraglottic	26	1	12	13		
subglottic	3	1	1	1		
**Total**	134	39	59	36		

**Note:** a:correlation coefficient,b: median age;pT=pathologic tumor;pN=pathologic node; pN+=pN1/ pN2/pN3; pTNM=pathologic tumor/node/ metastasis;PTK6=protein tyrosine kinase6.

### Relationship between PTk6 expression and LSCC Patients’ survival

To explore the relationship between PTK6 expression and LSCC patients’ survival, we applied Kaplan-Meier analysis. Among the 134 patients with LSCC, 102 patients were alive and 32 were died at the last clinical follow-up, and the median observation period was 79 months (range, 0.6 to 112 months). The 3-year DFS and OS for the entire cohort of patients were 81.5% and 84%, respectively, and 5-year DFS and OS were 77.2% and 79.4%, respectively.

**Figure 3:**
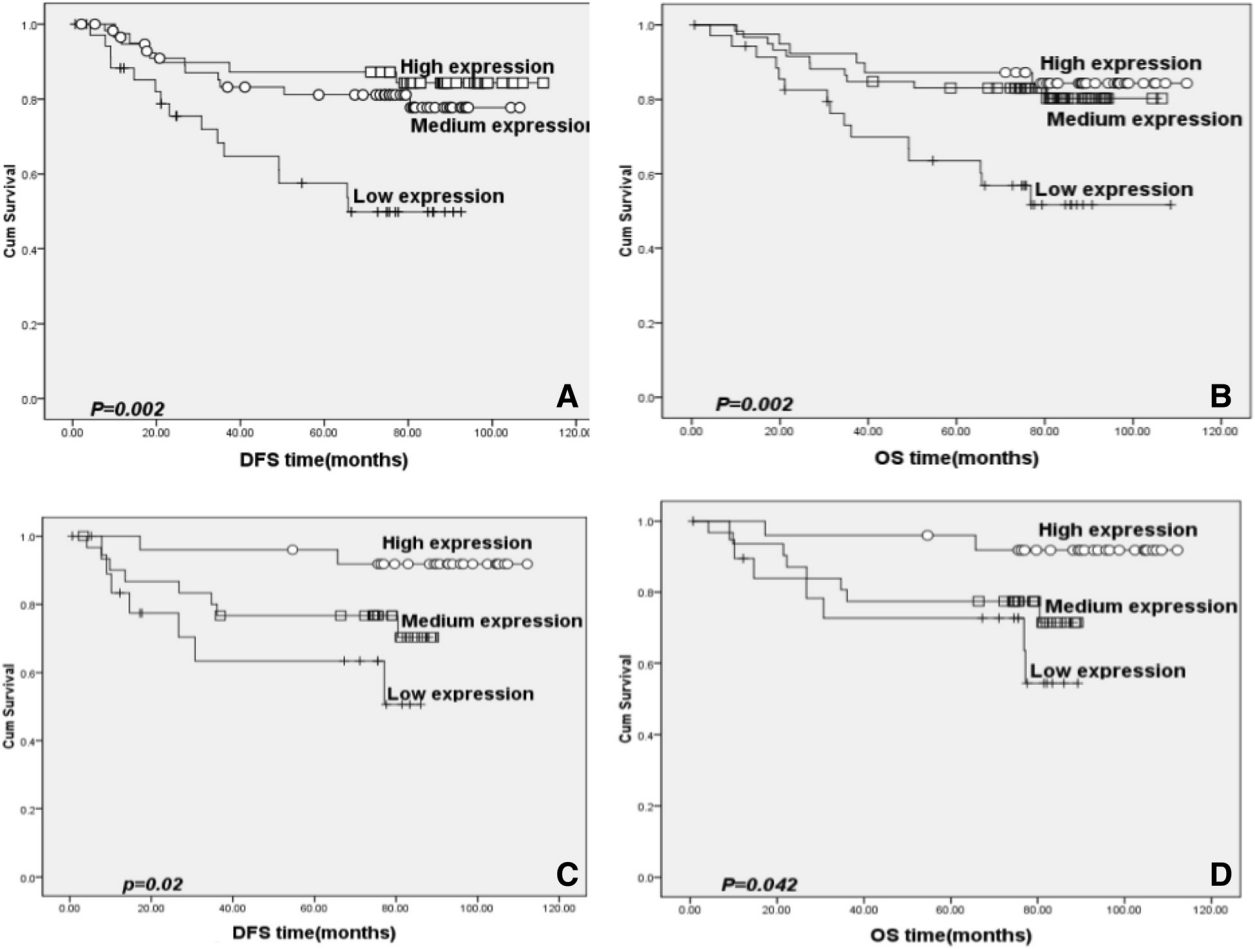
**The Kaplan-Meier survival analysis in different PTK6 expression levels of laryngeal squamous cell carcinoma (LSCC) patients. A **and **B**: The survival curve of high, medium and low PTK6 expression LSCC patients in the primary LSCC category (**A**: DFS, *P* = 0.002; **B**: OS *P* = 0.002); **C **and **D**: The survival curve of high, medium and low PTK6 expression LSCC patients in adjacent paracancerous laryngeal epithelial sample (**C**: DFS, *P* = 0.02; **D**: OS, *P* = 0.042).

In the primary LSCC category, median DFS (disease free survival) of high, medium and low PTK6 expression patients were 88.5 months, 74.5 months and 49.0 months (log-rank test, *P* = 0.002; Figure 3A), respectively; Similarly, median OS (overall survival) of high, medium and low PTK6 expression patients were 88.5 months, 76.3 months and 65.7 months (log-rank test, *P* = 0.002; Figure 3B), respectively, suggesting that PTK6 expression was closely associated with DFS and OS for LSCC patients (*P* < 0.001). The cumulative 5-year DFS and OS of high, medium and low PTK6 expression patients were 87.2% & 87.2%, 81.1% & 83.0%, and 57.5% and 63.6%, respectively.

In addition, we analyzed the patients’ survival in terms of PTK6 expression in adjacent noncancerous tissues and found that, median DFS of high, medium, and low PTK6 expression patients were 92.6 months, 75.6 months and 48.5 months (log-rank test, *P* = 0.020, Figure 3C), respectively; and median OS of high, medium and low PTK6 expression patients were 92.9 months, 78.9 months and 74.6 months (log-rank test, *P* = 0.042, Figure 3D), respectively, indicating the expression of PTK6 in adjacent noncancerous tissues was also strongly correlated with patients’ survival.

Furthermore, we stratified patients’ survival by pN0 status and demonstrated that the 3- year and 5-year DFS of high, medium and low PTK6 expression patients were 94.4% & 91.7%, 85.3% & 83.1%, and 81.7% &71.5%, (log-rank test, *P* = 0.028), respectively; Likewise, the 3- and 5-year OS of high, medium and low PTK6 expression patients were 97.2% & 91.7%, 86.5% & 84.6%, and 82.9% & 74.2% (log-rank test, *P* = 0.049), respectively. If stratified by surgery treatments, the 3- and 5-year DFS of high, medium and low PTK6 expression patients were 94.4% & 91.7%, 85.3% & 83.1%, and 81.7% &71.5% (log-rank test, *P* = 0.005), respectively; Similarly, the 3- and 5-year OS of high, medium and low PTK6 expression patients were 96.4% & 96.4%, 91.1% & 91.1%, and 76.5% & 63.7% (log-rank test, *P* < 0.0001), respectively.

To determine whether PTK6 expression could serve as an independent prognostic factor, we examined DFS and OS using the Cox proportional hazards model. PTK6 expression and several other clinicopathological factors, such as treatments, pathology TNM stage, and pN status were found to be associated with DFS and OS by univariate analysis. All variables were further analyzed by multivariate analysis, which revealed that PTK6 expression and pN status were independent significant prognostic factors for DFS and OS (Table 2). Additionally, the RRs (relative risks) showed that high PTK6 expression had lower risk than low PTK6 expression, and that no cervical lymph node metastasis had lower risk than lymph node metastasis.

**Table 2:** Univariate and multivariate cox regression analysis for disease-free survival and overall survival in patients with laryngeal squamous cell carcinoma

	**Disease-free survival**				**Overall survival**			
	**Univariate analysis**		**Multivariate analysis**		**Univariate analysis**		**Multivariate analysis**	
Factors	RR(95% CI)	*P *value^a^	RR(95% CI)	*P *value^a^	RR(95% CI)	*P *value^a^	RR(95% CI)	*P *value^a^
Age^b^	0.997(0.492 -2.019)	0.397			1.026(0.507 -2.078)	0.337		
Sex^c^	0.997(0.492 -2.020)	0.560			1.026(0.507 -2.079)	0.457		
Tumor location^d^	0.997(0.492 -2.021)	0.053			1.026(0.507 -2.080)	0.085		
Pathology grade^e^	0.997(0.492 -2.022)	0.003	1.074(0.643 -1.795)	0.786	1.026(0.507 -2.081)	0.003	1.055(0.632 -1.763)	0.837
pT stage^f^	0.997(0.492 -2.023)	0.013	1.074(0.643 -1.796)	0.434	1.026(0.507 -2.082)	0.013	1.055(0.632 -1.764)	0.291
Pathology TNM status^g^	0.997(0.492 -2.024)	0.002	1.074(0.643 -1.797)	0.886	1.026(0.507 -2.083)	0.003	1.055(0.632 -1.765)	0.848
Treatment^h^	0.997(0.492 -2.025)	0.005	1.074(0.643 -1.798)	0.167	1.026(0.507 -2.084)	0.015	1.055(0.632 -1.766)	0.164
pN stage^I^	0.997(0.492 -2.026)	<0.001	1.074(0.643 -1.799)	0.04	1.026(0.507 -2.085)	<0.001	1.055(0.632 -1.767)	0.03
PTK6^k^ expression	0.997(0.492 -2.027)	0.002	1.074(0.643 -1.800)	0.029	1.026(0.507 -2.086)	0.002	1.055(0.632 -1.768)	0.038

a Cox proportional hazards model;b ≤58 years versus > 58 years;c Male versus female;d glottic versus supraglottic versus subglottic;e well versus mediate versus poor differential grade; f pT1/pT2 versus pT3 /pT4; g pathological I/II versus III/IV; h surgery versus surgery+ radiotherapy versus surgery+chemotherapy; I pN0 versus pN+; k High versus medium versus low expression; RR=relative risk; CI=confidence interval; pT= pathologic tumor; pN=pathologic node.

## Discussion

In the present study, we showed that PTK6 expression was decreased in primary LSCC tissues compared to the adjacent noncancerous tissues at both protein and mRNA levels. It was the first time to report that low expression of PTK6 was correlated with late pathology nodal stage, advanced pTNM stages (III and IV), and poor differentiated LSCC. LSCC patients with low expression of PTK6 had reduced DFS and OS than those with high expression of PTK6, suggesting the PTK6’s clinical value in assessing the prognosis of LSCC patients. In adjacent paracancerous laryngeal epithelial sample, there were significant survival differences among high, medium and low PTK6 expression patients. Moreover, patients with medium PTK6 expression had worse DFS and OS than those with high expression of PTK6, suggesting that PTK6 loss from non-cancerous tissue could be an early event in transformation. Interestingly, in the subgroup analysis, including pN0 status and treatments, high PTK6 expression displayed a statistically favored significant effect on survival. Taken together, these findings indicated that PTK6 might play an important role in the tumor differentiation and progression of LSCC.

Clinically, current methods used to determine the prognosis of LSCC patients were mainly dependent on T classification, lymph node status, distant metastasis, clinical staging, differentiation grade and metastasis [10-15]. In current study, the univariate and multivariate Cox proportional hazard analysis revealed pathology lymph node metastasis was associated with a high risk for cancer-related death (Table 2). In practice, nodal staging was one of the most important prognostic factors in LSCC patients [11,16,17]. However, many patients with the same clinicopathological factors had distinct outcomes, indicating that clinicopathological factors may be insufficient to fully prognosticate survival. Therefore, it is important to explore more prognostic factors, such as molecular biomarkers, to prognosticate survival and to help LSCC patients decide on treatment options. Although several interaction partners of PTK6 have been identified in cellular cultures, and positive PTK6 expression in breast cancer tissue is associated with better prognosis survival [18], the function of PTK6 as well as its function in LSCC development and survival prognosis remained unclear.

PTK6 possessed an amino-terminal SH3 domain, a central SH2 domain, and a carboxyterminal tyrosine kinase domain [19,20]. Although PTK6 structurally resembled Src-family tyrosine kinases, PTK6 lacked an N-terminal myristoylation site which is required by Src kinases for membrane localization [2]. PTK6 activity is positively regulated by autophosphorylation at Y342 within the PTK6 kinase domain, and negatively regulated by Y447 phosphorylation [21], which might explain the paradoxical patterns of expression and various roles of PTK6 in different tissues [3]. Several studies [22-24] demonstrated the variable expression level and cellular localization of PTK6 among different tissues. Petro et al. [3] found that PTK6 expression mainly in cytosol in moderately differentiated oral squamous cell carcinoma cells and in perinucleus regions in poorly differentiated cells, with a correlation between reduced PTK6 expression and decreased differentiation. Derry et al. [22] also found that PTK6 expression was mainly expressed in nuclei in well differentiated prostate carcinomas, but mainly in the cytosol in poorly differentiated and highly tumorigenic prostate carcinoma PC3 cells. In our study, we found that high expression of PTK6 was associated with well differentiation of LSCC, suggesting a possible role for PTK6 in differentiation of laryngeal squamous cells. However, the molecular mechanism by which PTK6 participates in differentiation remains unclear.

Overexpression of PTK6 alone sensitizes mammary epithelial cells to mitogenic effects of EGF (epidermal growth factor) [8], and its coexpression with epidermal growth factor receptor (EGFR)-related receptor ErbB3 (ErbB3, also known as human epidermal growth factor receptor3 (Her3)) markedly enhances EGF signaling via AKT and PI-3 kinase [25]. Different ErbB receptor ligands, including EGF and heregulin, stimulated PTK6 activity [8,25-27]. Furthermore, inhibition of PTK6 expression in breast tumor cells resulted in a moderate decrease in cellular proliferation using small inhibitory RNA [28]. These data collectively have leaded studies to validate PTK6 as a therapeutic target in breast cancer [20]. Moreover, published data demonstrated that the expression of a mutant PTK6, which abrogates the kinase activity by a mutation in the ATP-binding site (K-M mutation), exhibited reduced cell motility compared to wild-type PTK6 [27]. Ma et al. [29] identified PTK6 as tumor suppressor in esophageal carcinoma, and they found that the expression of PTK6 was down-regulated in the tumor due to promoter hypermethylation and histone deacetylation. Future studies will explore the function and downstream signaling pathways of PTK6 in the development of LSCC and investigate a possible crosstalk between PTK6 and EGF pathway.

## Conclusion

In our study, the PTK6 protein expression was higher in normal paracancerous tissue than in LSCC tissues by western blotting and RT-PCR, and the patients with high expression of PTK6 had better DFS and OS than those with low expression patients. Together, these studies suggested the potential role of for PTK6 in cell migration and tumorigenesis, implying that the overexpression of PTK6 may serve as a potential therapy for LSCC patients.

In conclusion, we showed PTK6 high expression in a cohort of 134 LSCC cases with completely resection is a valuable independent prognostic predictor. However, how PTK6 expression in this malignant tumor is modulated and functions still needs more investigations. Future studies on the cellular function and interaction pathways of PTK6 and its potential role in the development of LSCC cancer will be helpful for further understanding of the progress of this malignant tumor and improving clinical therapies.

## Competing interests

The authors declare that they have no competing interests.

## Authors’ contributions

XUE-KUI LIU and XIN-RUI ZHANG carried out the molecular genetic studies, participated in the sequence alignment, the design of the study and drafted the manuscript. QIAN ZHONG and MAN-ZHI LI carried out the immunoassays. ZHI-MIN LIU and DI WU participated in literature research; ZHI-RUI LIN participated in the statistical analysis. MU-SHENG ZENG conceived of the study, and participated in its design and coordination and helped to draft the manuscript. All authors read and approved the final manuscript.

## Funding

National Natural Science Funds for Distinguished Young Scholar (81025014), the National Natural Science Foundation of China (81161120408 and 91019015).
